# Pattern of the heart rate performance curve in maximal graded treadmill running from 1100 healthy 18–65 Years old men and women: the 4HAIE study

**DOI:** 10.3389/fphys.2023.1178913

**Published:** 2023-05-30

**Authors:** Philipp Birnbaumer, Tomas Dostal, Lukas Cipryan, Peter Hofmann

**Affiliations:** ^1^ Exercise Physiology, Training and Training Therapy Research Group, Institute of Human Movement Science, Sport and Health, University of Graz, Graz, Austria; ^2^ Department of Human Movement Studies & Human Motion Diagnostic Centre, The University of Ostrava, Ostrava, Czech Republic

**Keywords:** exercise prescription, exercise intensity, thresholds, performance diagnostics, heart rate turnpoint

## Abstract

**Introduction:** The heart rate performance curve (HRPC) in maximal incremental cycle ergometer exercise demonstrated three different patterns such as downward, linear or inverse versions. The downward pattern was found to be the most common and therefore termed regular. These patterns were shown to differently influence exercise prescription, but no data are available for running. This study investigated the deflection of the HRPC in maximal graded treadmill tests (GXT) of the 4HAIE study.

**Methods:** Additional to maximal values, the first and second ventilatory thresholds as well as the degree and the direction of the HRPC deflection (k_HR_) were determined from 1,100 individuals (489 women) GXTs. HRPC deflection was categorized as downward (k_HR_ < −0.1), linear (−0.1 ≤ k_HR_ ≤ 0.1) or inverse (k_HR_ > 0.1) curves. Four (even split) age- and two (median split) performance-groups were used to investigate the effects of age and performance on the distribution of regular (= downward deflection) and non-regular (= linear or inverse course) HR curves for male and female subjects.

**Results:** Men (age: 36.8 ± 11.9 years, BMI: 25.0 ± 3.3 kg m^−2^, VO_2max_: 46.4 ± 9.4 mL min^−1^. kg^−1^) and women (age: 36.2 ± 11.9 years, BMI: 23.3 ± 3.7 kg m^−2^, VO_2max_: 37.4 ± 7.8 mL min^−1^. kg^−1^) presented 556/449 (91/92%) downward deflecting, 10/8 (2/2%) linear and 45/32 (7/6%) inverse HRPC´s. Chi-squared analysis revealed a significantly higher number of non-regular HRPC´s in the low-performance group and with increasing age. Binary logistic regression revealed that the odds ratio (OR) to show a non-regular HRPC is significantly affected by maximum performance (OR = 0.840, 95% CI = 0.754–0.936, *p* = 0.002) and age (OR = 1.042, 95% CI = 1.020–1.064, *p* < 0.001) but not sex.

**Discussion:** As in cycle ergometer exercise, three different patterns for the HRPC were identified from the maximal graded treadmill exercise with the highest frequency of regular downward deflecting curves. Older subjects and subjects with a lower performance level had a higher probability to show a non-regular linear or inverted curve which needs to be considered for exercise prescription.

## 1 Introduction

**FIGURE 1:**
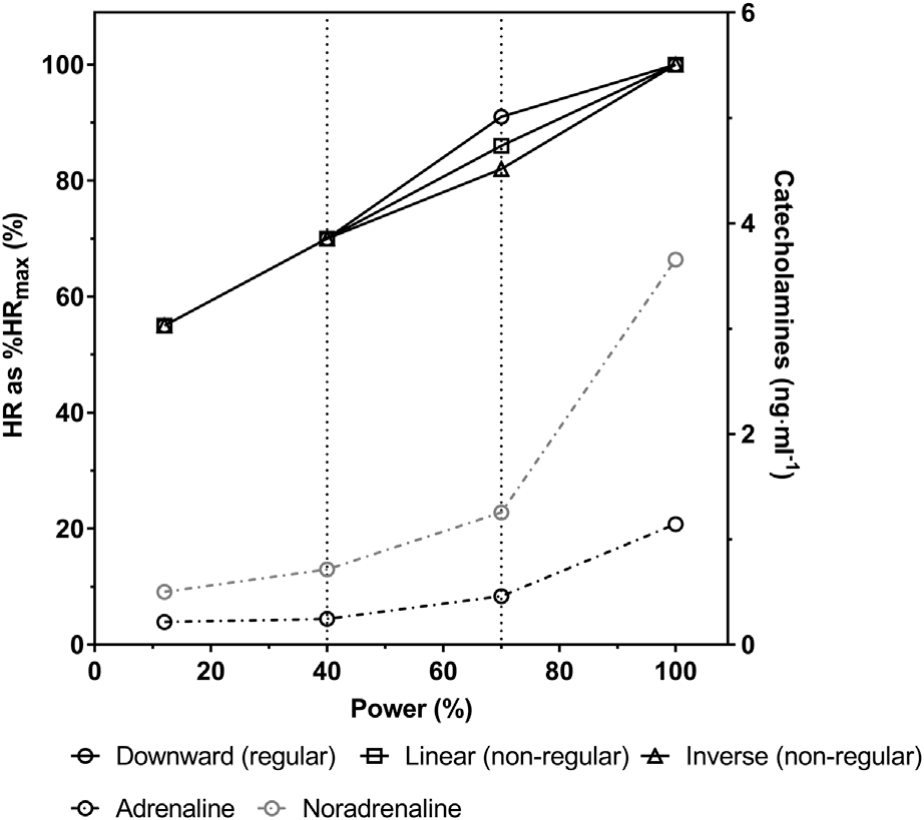
Schematic 3—phase model of the heart rate performance curve (HRPC) during incremental cycle ergometer exercise with regular downward deflection and non-regular linear or inverse course (solid black lines) as well as plasma adrenaline and noradrenaline concentrations (dashed and dash-dotted black and grey lines, respectively) (modified from Hofmann et al., 1997; Pokan et al., 1995).

The heart rate performance curve (HRPC) denotes the heart rate increase during incremental exercise, which demonstrated three different patterns such as downward, linear or inverse versions (Brooke et al., 1968; Hofmann et al., 2001; Hofmann et al., 1997). Based on the actual standard model of a three phase-two threshold model of energy supply (Binder et al., 2008; Mezzani, 2017; Jamnick et al., 2020) three different HRPC patterns for cycle ergometer exercise were demonstrated. The classification was based on the intersection angle of two regression lines between the first lactate or ventilatory threshold and maximum exercise. The most frequent and common pattern is s-shaped, characterized by a small HR increase in phase I up to the first intensity threshold, a linear increase proportional to the workload in phase II up to the second intensity threshold and a downward deflection above the second intensity threshold approaching maximum exercise intensities (Figure 1). This pattern has therefore been defined the regular pattern. Non-regular patterns were defined as HRPC´s presenting a blunted HR increase in the second phase with a linear time course, presenting no deflection at all or even a disproportionate increase respectively inverted (upward) deflection above the second threshold approaching maximal exercise intensities (Figure 1) (Hofmann et al., 1997).

Hofmann et al. (1997) showed an unequal distribution of the HRPC patterns in maximum incremental cycle ergometer exercise in a large age homogenous group of healthy young trained male individuals. An S-shaped time course of the HRPC was the main pattern in about 86% of the participants and it was therefore considered and termed regular HRPC. However, a significant number of curves termed non-regular were found to present a linear time course (6%) or even an inverted (8%) one. Recently, we showed in a large heterogenous group of healthy male and female subjects, that the distribution of the HRPC was dependent on age, sex and the performance level (Birnbaumer et al., 2020). Additionally, similar analyses in a large group of male and female patients receiving beta-receptor antagonist treatment supported the already known influence of this medication on the HRPC patterns (Hofmann et al., 2005; Birnbaumer et al., 2021). As shown before, beta-receptor antagonist medication changed regular curves into non-regular curves and already non-regular curves stayed non regular (Hofmann et al., 2005). Men compared to women as well as aging, a low performance level and the use of beta-receptor antagonist medication were shown to be significantly related with a higher number of non-regular curves (Birnbaumer et al., 2020; Birnbaumer et al., 2021). The main cause for non-regular HRPC´s was suggested to be a reduced ß1-adrenoceptor sensitivity (Hofmann et al., 2005) supported by results from Moser et al. (2018) and confirmed by our latest results (Birnbaumer et al., 2020; Birnbaumer et al., 2021).

In practice the deflection of the HRPC is used to determine performance markers like the heart rate turn point (HRTP) which is based on findings from Conconi et al. (1982) and was suggested to be equivalent to the second lactate or ventilatory threshold (Hofmann et al., 1994; Bodner and Rhodes, 2000; Binder et al., 2008). The HRTP allows an accurate noninvasive individualized prescription of exercise intensity and its determination was shown to be independent from the direction of the deflection but is not possible in HRPC´s with a linear time course (Hofmann et al., 1997). However, the type of the HRPC deflection has been shown to cause differences in the HR value at HRTP as well as in its percentage in relation to HR_max_ (Hofmann et al., 2001) which has some main implications on exercise prescription. Furthermore, the degree of the HRPC deflection was shown to be effected by regular endurance training in cardiac rehabilitation which led to a normalization of the curves (Heber et al., 2019). Additionally, glycemic control in type 1 diabetes patients was significantly associated to the k_HR_ where higher HbA1c values were associated with more pronounced non-regular curves (Moser et al., 2018). The deflection of the HRPC is therefore suggested to give additional information about the health and performance status of a subject. If running tests show HRPC patterns comparable to those of cycling, an effect independent of the type of exercise may be hypothesized.

Up to now most investigations on the HRPC deflection applied cycle ergometer exercise and there are only few data available for running (Rosic et al., 2011) or other activities. Therefore, the aim of this study was to investigate the distribution of the HRPC patterns during maximal graded treadmill exercise and the possible influences of sex, age and performance in a large heterogeneous sample of healthy male and female subjects of the 4HAIE study (Cipryan et al., 2020). A secondary aim was to establish reference data of submaximal and maximal performance and spirometric variables in maximal graded treadmill tests from 1 100 healthy active and inactive men and women aged 18–65 years.

## 2 Methods

### 2.1 Participants

The 4HAIE (Healthy Aging in Industrial Environment) study addresses the effects of selected environmental and lifestyle risk factors on health and aging of the population in an industrial region. In this single-center, prospective, longitudinal and multidisciplinary cohort study 1,314 (608 female) adults (age 18–65 years) living in a high (750) or low (564) air-polluted area of the Czech Republic participated and were either regular runners (747) or inactive (567) individuals (Cipryan et al., 2020). Eligible participants had to be free from acute health conditions preventing from physical activity, acute disease, pregnancy, radiological examination within the last 7 days, artificial pacemaker, radioactive, surgical or any other device/implant, insulin pump and smoking. The main purpose the 4HAIE study was to monitor whether physical activity in polluted air and some other characteristics (psychosocial, biomechanical, physiological and somatometric) can affect health, mental wellbeing and quality of life.

This analysis includes anthropometric as well as performance, HR and spirometric data from maximal graded exercise test (GXT) of 1,100 participants from the 4HAIE data set. The original number was reduced by 118 participants who did not perform a GXT in the 4HAIE screening and by further 96 data sets in which the GXT duration was too short to correctly determine thresholds and/or the HR deflection, the HR data had a poor quality (missing values), HR data were not plausible or there was no HR data available at all.

### 2.2 Ethics statement

The study was approved by the Ethics Committee of the University of Ostrava (3/2018). Each participant was provided a detailed information sheet and a written informed consent was obtained.

### 2.3 Graded exercise test (GXT)

Participants performed a laboratory GXT until volitional exhaustion on a motorized treadmill (RL 2500E, Rodby Innovation AB, Sweden) to determine maximal oxygen uptake (V̇O_2max_) and maximum running speed (v_max_) as well as the first and second ventilatory thresholds (VT_1_ and VT_2_). The GXT started with a 3 min familiarization phase of walking at 5.0 km h^-1^. Subsequently, speed was increased by 1.0 km h^-1^ every minute starting at 6.0 km h^-1^ (inclination remained at 1%). Expired air was continuously measured during the GXT with a breath-by-breath system (Blue Cherry, Geratherm Medical AG, Germany). The highest average O_2_ consumption measured over a 30 s period was used to determine V̇O_2max_. VT_1_ was determined as the first increase in ventilation (V̇E) accompanied by a minimum of the oxygen equivalent (V̇E/V̇O_2_) without an increase in the carbon dioxide (CO_2_) equivalent (V̇E/V̇CO_2_). VT2 was determined as the second sharp increase in V̇E accompanied by an increase in both V̇E/V̇O_2_ and V̇E/V̇CO_2_ (Binder et al., 2008). All thresholds were detected by multi linear regression analyses *via* Vienna CPX-Tool (University of Vienna, Austria). HR was measured using a chest belt monitor (Polar Electro H9, Kempele, Finland). All exercise tests were conducted in the afternoon, at least 3 h after the participants last meal in a thermally controlled laboratory (21°C, 40% relative humidity). Each participant was advised not to participate in any vigorous activity 24 h prior to the test.

### 2.4 HRPC deflection

The degree and the direction of the HRPC deflection (k_HR_) were calculated automatically with the Vienna CPX-Tool from the slopes of two tangents of a second-degree polynomial function between VT_1_ and v_max_. Based on the k_HR_ values all HRPC´s were categorized as downward deflecting HRPC´s for k_HR_ < −0.1, inverse HRPC´s for k_HR_ > 0.1 and HRPC with a linear course for −0.1≤ k_HR_ ≤ 0.1 according to Pokan et al. (1993). Differences in the application of the k_HR_ equation resulted in differences in the algebraic sign compared to the original publication by Pokan et al., however not affecting the categorization of the HRPC. For analysis, HRPC´s with a linear course and HRPC presenting an upward deflection were combined and both categorized as non-regular HRPC´s and curves presenting a downward deflection were categorized as regular HRPC, according to our earlier publications (Hofmann et al., 2001; Birnbaumer et al., 2021).

### 2.5 Statistical analysis

Data are shown as mean ± standard deviation. To support the assumption of normally distributed data, parametric analysis and QQ-plots were used. Differences in the anthropometric and performance data between men and women were tested by independent-sample t tests. To examine the effects of age, four age-groups were defined aiming for an equal number of participants. Analysis of variance (ANOVA) was used to determine differences in anthropometric and performance data between age-groups. If significant effects were found, *post hoc* evaluations were completed using Bonferroni tests. To examine performance effects, a low- and high-performance-group by median split of v_max_ were defined. Independent-sample t tests were used to analyze differences between performance-groups for anthropometric and performance data. Chi-squared tests were applied to analyze the distribution of HRPC patterns depending on sex, age- and performance-groups. Post hoc analysis of the Chi-squared test for age-groups were done by transformation of the standardized residuals into a Chi-squared value and comparison of these values with the *p*-value of a Bonferroni correction. Additionally, a binary logistic regression analysis was calculated to evaluate the relationship between the HRPC pattern and the factors sex (dichotomous), age (continuous) and performance (v_max_) (continuous). All statistical analyses were performed in SPSS 26 (IBM Corporation, United States). Graphical representations were created with Prism 8.0 (GraphPad, United States). Statistical significance was considered as *p* < 0.05.

## 3 Results

### 3.1 Performance data from GXT

**FIGURE 2:**
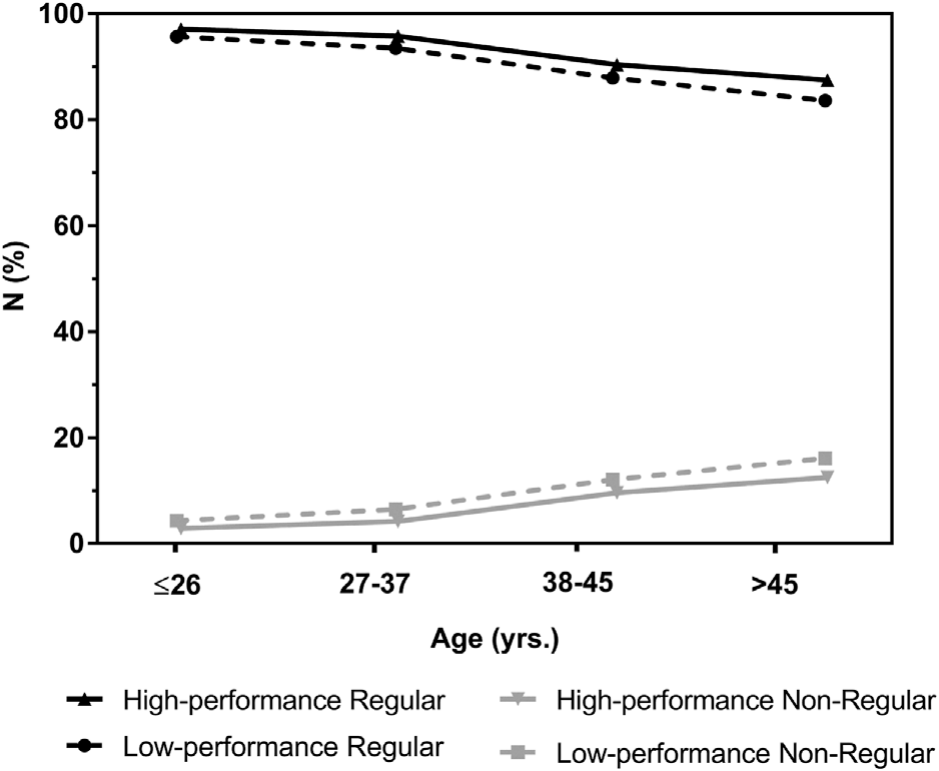
Heart rate (HR) at the second ventilatory threshold (VT_2_) as percentage of maximum heart rate (%HR_max_) in participants with a regular or non-regular deflection of the heart rate performance curve in maximal incremental treadmill exercise tests. Data are presented as box plot showing the median, upper and lower quartile as well as minimum and maximum values.

Descriptive characteristic for men and women as well as their absolute and relative performance markers from GXT are shown in Tables 1, 2. Mean absolute maximal and submaximal performance, HR and V̇O_2_ values as well as relative performance, HR and V̇O_2_ values at VT_1_ and VT_2_ were significantly different between men and women. Mean V̇O_2max_ was 46.4 ± 9.4 mL min^-1^. kg^-1^ in men and 37.4 ± 7.8 mL min^-1^. kg^-1^ in women and was significantly lower in the low-compared to the high-performance groups in both sexes. Compared to the youngest age-group, V̇O_2max_ was significantly lower in the age-groups >26 years in men and >37 years in women. Comparable, v_max_ and v at VT_1_ and VT_2_ were significantly lower in the low-compared to the high-performance group as well as in the age groups >37 years in men and women (except for v_VT1_ in women which was significantly different >45 years). Performance, HR and VO_2_ values at VT_1_ and VT_2_ as a percentage of maximum were in some cases significantly different between performance- and age groups, but varied less compared to absolute values (Tables 1, 2). HR at VT_2_ as percentage of HR_max_ (%HR_max_) was significantly higher at 91.3% ± 3.3% in participants with a regular HRPC compared to 87.9% ± 5.6% in subjects with non-regular HRPC´s. When comparing HRTP to VT_2_ a significant difference was found for %HR_max_, however, mean values were very similar with a difference of only 0.36%. Figure 2 shows %HR_max_ values at VT_2_ in a box plot.

**TABLE 1:** Maximal as well as absolute and relative submaximal performance markers of the maximum graded treadmill tests in men.

Men							
		Performance-level		Age-groups			
	Total	Low	High	≤26 years	27–37 years	38–45 years	>45 years
**N** ( )	611	317	294	149	161	150	151
**Age** (yrs.)	36.8 ± 11.9	39.9 ± 12.7	33.4 ± 10.0*	21.5 ± 2.6	31.8 ± 2.9*	41.4 ± 2.1*	52.4 ± 5.5*
**Weight** (kg)	81.5 ± 11.8	85.9 ± 12.1	77.0 ± 9.6*	75.7 ± 12.0	82.5 ± 11.2*	83.9 ± 10.5*	83.7 ± 11.7*
**BMI** (kg.m^-2^)	25.0 ± 3.3	26.3 ± 3.5	23.6 ± 2.3*	23.3 ± 3.1	25.1 ± 3.2*	25.4 ± 2.8*	26.1 ± 3.4*
**v**_**max**_ (km.h^-1^)	14.8 ± 2.4	13.0 ± 1.7	16.7 ± 1.2*	15.6 ± 2.0	15.5 ± 2.1	14.8 ± 2.0*	13.2 ± 2.4*
**HR**_**max**_ (bpm)	182.5 ± 13.4	180.7 ± 14.6	184.3 ± 11.7*	194.0 ± 8.2	186.0 ± 10.0*	178.4 ± 10.6*	170.5 ± 11.5*
V̇**O**_**2max**_ (mL.min^-1^.kg^-1^)	46.4 ± 9.4	39.9 ± 7.4	53.4 ± 5.8*	51.3 ± 7.7	48.5 ± 8.4*	45.4 ± 8.4*	40.3 ± 9.6*
**v**_**VT1**_ (km.h^-1^)	8.1 ± 1.0	7.4 ± 0.6	8.8 ± 0.7*	8.4 ± 1.0	8.3 ± 1.0	8.0 ± 0.7*	7.5 ± 0.9*
**v**_**VT2**_ (km.h^-1^)	11.4 ± 1.7	10.1 ± 1.3	12.7 ± 0.9*	12.0 ± 1.5	11.9 ± 1.6	11.4 ± 1.5*	10.2 ± 1.8*
**HR**_**VT1**_ (bpm)	138.1 ± 14.9	136.5 ± 16.2	139.8 ± 13.4*	148.3 ± 12.3	140.2 ± 12.9*	135.4 ± 13.6*	127.3 ± 13.1*
**HR**_**VT2**_ (bpm)	165.8 ± 13.5	164.4 ± 15.2	167.3 ± 11.3*	177.3 ± 8.9	169.6 ± 10.2*	162.1 ± 11.0*	154.0 ± 11.8*
V̇**O**_**2VT1**_ (L.min^-1^)	2.3 ± 0.4	2.1 ± 0.4	2.5 ± 0.4*	2.3 ± 0.5	2.3 ± 0.4	2.3 ± 0.4	2.1 ± 0.4*
V̇**O**_**2VT2**_ (L.min^-1^)	3.1 ± 0.5	2.9 ± 0.5	3.4 ± 0.5*	3.2 ± 0.5	3.3 ± 0.5	3.2 ± 0.5	2.9 ± 0.5*
**v as % v**_**max**_							
**VT**_**1**_ (%)	54.2 ± 3.8	55.9 ± 3.6	52.6 ± 3.2*	53.7 ± 3.7	53.9 ± 3.7	53.7 ± 3.1	55.8 ± 4.2*
**VT**_**2**_ (%)	77.1 ± 2.8	77.8 ± 2.7	76.3 ± 2.8*	76.9 ± 2.5	76.6 ± 3.3	77.2 ± 2.5	77.5 ± 2.8
***HR as % HR***_***max***_							
**VT**_**1**_ (%)	75.4 ± 5.6	74.9 ± 6.1	75.8 ± 5.1*	76.3 ± 5.5	75.1 ± 5.6	75.4 ± 5.4	74.6 ± 5.9
**VT**_**2**_ (%)	90.8 ± 2.7	90.8 ± 3.1	90.8 ± 2.0	91.3 ± 2.3	90.9 ± 2.5	90.8 ± 2.6	90.2 ± 3.1
V̇ ***O***_***2***_ ***as %*** V̇ ***O***_***2max***_							
**VT**_**1**_ (%)	59.5 ± 7.0	59.2 ± 7.9	59.8 ± 6.1	58.2 ± 6.9	58.7 ± 6.5	60.1 ± 7.4	61.3 ± 7.0*
**VT**_**2**_ (%)	82.9 ± 5.8	84.3 ± 6.0	81.3 ± 5.1*	81.5 ± 5.2	82.0 ± 5.7	82.9 ± 5.9	85.1 ± 5.7*

Significantly different compared to low-fit respectively to the age-group ≤26 yrs (*p* < 0.05).

**TABLE 2:** Maximal as well as absolute and relative submaximal performance markers of the maximum graded treadmill tests in women.

Women							
		Performance-level		Age-groups			
	Total	Low	High	≤26 years	27–37 years	38–45 years	>45 years
**N** ( )	489	251	238	140	112	118	119
**Age** (yrs.)	36.2 ± 11.9	40.1 ± 12.4	32.1 ± 9.8^a^	21.7 ± 2.5	31.9 ± 3.1^a^	42.0 ± 2.2^a^	51.7 ± 4.5^a^
**Weight** (kg)	65.8 ± 11.3	70.4 ± 11.7	61.0 ± 8.5	62.6 ± 10.1	65.0 ± 12.2	66.6 ± 11.6^a^	69.5 ± 10.1^a^
**BMI** (kg.m^-2^)	23.3 ± 3.7	25.0 ± 4.0	21.6 ± 2.4^a^	22.2 ± 3.3	22.8 ± 3.7	23.7 ± 3.9^a^	24.8 ± 3.6^a^
**v**_**max**_ (km.h^-1^)	12.1 ± 2.1	10.4 ± 1.2	13.9 ± 1.2^a^	13.0 ± 2.0	12.8 ± 2.0	12.1 ± 2.0^a^	10.6 ± 1.7^a^
**HR**_**max**_ (bpm)	180.5 ± 11.3	177.6 ± 11.4	183.4 ± 10.4^a^	190.3 ± 7.8	182.9 ± 8.4^a^	176.7 ± 8.7^a^	170.4 ± 8.8^a^
V̇**O**_**2max**_ (mL.min^-1^.kg^-1^)	37.4 ± 7.8	31.9 ± 5.4	43.2 ± 5.3^a^	40.5 ± 6.8	39.5 ± 7.5	37.2 ± 7.5^a^	32.0 ± 6.6^a^
**v**_**VT1**_ (km.h^-1^)	7.2 ± 0.7	6.6 ± 0.4	7.7 ± 0.6^a^	7.4 ± 0.8	7.4 ± 0.7	7.2 ± 0.6	6.8 ± 0.6^a^
**v**_**VT2**_ (km.h^-1^)	9.5 ± 1.6	8.2 ± 1.0	10.8 ± 1.0^a^	10.1 ± 1.5	10.0 ± 1.5	9.5 ± 1.5^a^	8.4 ± 1.3^a^
**HR**_**VT1**_ (bpm)	141.8 ± 14.5	137.9 ± 14.8	144.5 ± 13.7^a^	149.5 ± 12.9	142.5 ± 13.6^a^	139.4 ± 12.7^a^	130.7 ± 12.7^a^
**HR**_**VT2**_ (bpm)	166.9 ± 12.5	163.8 ± 13.6	170.1 ± 10.4^a^	177.2 ± 8.6	169.3 ± 8.7^a^	163.6 ± 9.8^a^	155.4 ± 11.1^a^
V̇**O**_**2VT1**_ (L.min^-1^)	1.6 ± 0.3	1.5 ± 0.3	1.7 ± 0.3^a^	1.6 ± 0.3	1.6 ± 0.3	1.6 ± 0.3	1.5 ± 0.3
V̇**O**_**2VT2**_ (L.min^-1^)	2.1 ± 0.4	1.9 ± 0.3	2.3 ± 0.3^a^	2.1 ± 0.4	2.2 ± 0.4	2.1 ± 0.4	1.9 ± 0.4^a^
**v as % v**_**max**_							
**VT**_**1**_ (%)	57.2 ± 4.0	60.0 ± 3.8	55.3 ± 2.9^a^	56.3 ± 3.8	56.9 ± 3.9	57.0 ± 3.6	59.7 ± 4.3^a^
**VT**_**2**_ (%)	78.5 ± 3.1	79.2 ± 3.2	77.8 ± 2.7^a^	77.9 ± 2.9	78.5 ± 2.8	78.6 ± 2.7	79.1 ± 3.7^a^
***HR as % HR***_***max***_							
**VT**_**1**_ (%)	78.1 ± 6.2	77.2 ± 6.7	78.7 ± 5.7^a^	78.6 ± 5.7	77.7 ± 6.5	79.0 ± 5.7	76.7 ± 6.7
**VT**_**2**_ (%)	92.4 ± 3.2	92.2 ± 3.9	92.7 ± 2.2^a^	93.0 ± 2.2	92.6 ± 2.3	92.7 ± 3.3	91.2 ± 4.4^a^
V̇ ***O***_***2***_ ***as %*** V̇ ***O***_***2max***_							
**VT**_**1**_ (%)	63.2 ± 8.3	62.7 ± 8.7	63.6 ± 8.0	61.7 ± 7.8	62.9 ± 8.7	64.1 ± 8.1	65.4 ± 8.6^a^
**VT**_**2**_ (%)	85.4 ± 5.8	86.1 ± 6.1	84.6 ± 5.5	84.1 ± 5.6	84.7 ± 6.3	85.9 ± 5.1	87.0 ± 6.0^a^

^a^ Significantly different compared to low-fit respectively to the age-group ≤26 yrs (*p* < 0.05).

### 3.2 HRPC deflection

Categorization of the HRPC by k_HR_ values revealed a number of 556 (91%) regular downward deflecting, 10 (2%) linear and 45 (7%) inverse HRPC´s in men and a number of 449 (92%) regular downward deflecting, 8 (2%) linear and 32 (6%) inverse HRPC´s in women. Merging of linear and inverse curves to non-regular curves resulted in a percentage of non-regular HRPC`s of 9% (men) and 8% (women).

Chi-squared analysis revealed no statistical difference in the distribution of the HRPC deflection pattern between men and women, but performance and age significantly affected the distribution of the HRPC pattern. Participants in the low-performance-group showed a significantly higher number of non-regular curves of 11% compared to the high-performance-group with only 5%. Analysis of the distribution depending on four age-groups revealed a significantly higher number of regular HRPC´s and lower number of non-regular HRPC´s in the youngest age group (≤26 years), but a significantly lower number of regular HRPC´s and higher number of non-regular HRPC´s as expected in the oldest age group (>45 years) (Table 3). Exclusion of linear HRPC´s from the analyses revealed similar results.

**TABLE 3:** Chi-Squared analysis of the distribution of regular and non-regular heart rate performance curves (HRPC`s) depending on sex, performance-group and age (age-groups).

		Cases	Regular HRPC	Non-regular HRPC	*p*-value		Cramers v
	**Total**	1,100	1,005	95			
	**%**	100%	91%	9%			
**Sex**	***Men***	611	556	55	0.630		**0.015**
	***%***	56%	91%	9%			
	***Women***	489	449	40			
	***%***	44%	92%	8%			
**Performance -Level**	***Low***	568	505	63	0.003		**0.090**
	***%***	52%	89%	11%			
	***High***	532	500	32			
	***%***	48%	94%	6%			
**Age-Groups**	***≤26 years***	289	279^b^	10^b^	<0.001		**0.171**
	***%***	26%	97%	3%			
	***27–37 years***	273	259	14			
	***%***	25%	95%	5%			
	***38–45 years***	268	239	29			
	***%***	24%	89%	11%			
	***>45 years***	270	228^c^	42^c^			
	***%***	25%	85%	15%			

^a^ Value = Asymptotic significance (two-sided).

^b^ Significantly higher number of regular HRPC´s but lower number of non-regular HRPC´s as expected.

^c^ Significantly lower number of regular HRPC´s but higher number of non-regular HRPC´s as expected.

**FIGURE 3:**
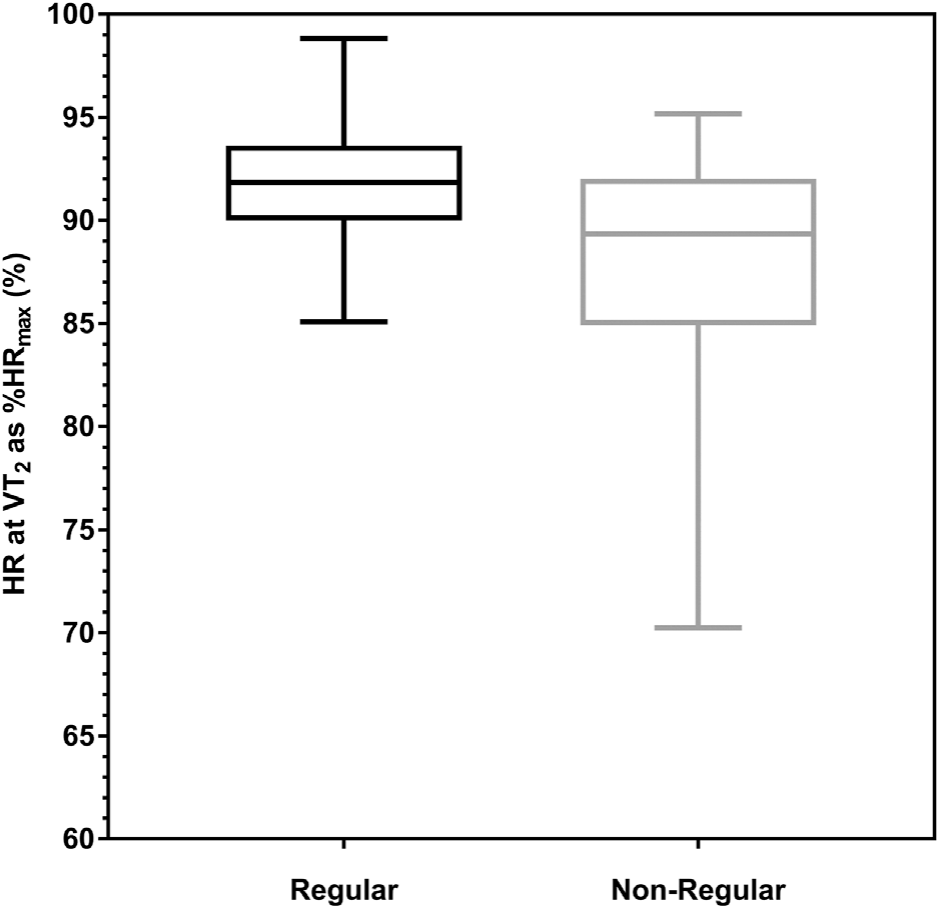
Deflection of the heart rate performance curve (HRPC) depending on age-groups in participants with low- and high- (50% percentile) performance levels. Significant effects of age and performance (v_max_) were shown in binary logistic regression [η2 (3, N = 1,104) = 50.637, *p* < 0.001, Nagelkerke´s R2 = 0.101].

Conducting a binary logistic regression analysis (η^2^ (3, N = 1,104) = 50.637, *p* < 0.001, Nagelkerke´s *R* ^2^ = 0.101) revealed that the odds ratio (OR) to show a non-regular HRPC was significantly affected by v_max_ (OR = 0.840, 95% CI = 0.754–0.936, *p* = 0.002) and age (OR = 1.042, 95% C I = 1.020–1.064, *p* < 0.001) but not sex. With every increase in v_max_ by 1 km h^-1^, the probability to show a non-regular curve decreases by 16%. However, with every 1-year increase of age the probability to show a non-regular curve increases by 4% (Figure 3).

## 4 Discussion

This study shows that the pattern of the HRPC in maximal incremental treadmill exercise also has three potential patterns, such as downward, inverse and linear. Categorizing the HRPC by k_HR_ according to the standard model (Pokan et al., 1993) revealed three different HRPC patterns comparable to cycle ergometer exercise (Hofmann et al., 1997; Birnbaumer et al., 2020). The most common was the downward deflecting pattern which is therefore defined “regular”. The distribution of regular and non-regular HRPC´s was dependent on age and performance but not sex as previously shown in the study with cycle ergometer (Birnbaumer et al., 2020). Older age and a low-performance level resulted in a higher number of non-regular HRPC´s (linear + inverse curves). With every year increase in age the odds to show a non-regular curve increased by 4.2% (95% CI 2.0%–6.4%), whereas every 1 km h^-1^ increase of v_max_ in the GXT reduced the odds to show a non-regular curve by 16.0% (95% CI 6.4%–24.6%).

### 4.1 HRPC deflection

Overall, the distribution of downward deflecting regular and non-regular inverse HRPC´s was comparable to results from earlier cycle ergometer exercise studies (Hofmann et al., 1997), although the number of linear curves was markedly lower in our treadmill exercise study (2%) compared to cycling (6%). The increasing number of non-regular curves with increasing age and decreasing performance has been shown in our earlier findings from cycle ergometer exercise in a large group of healthy individuals (Birnbaumer et al., 2020). In this study, non-regular curves were shown to increase in a linear trend from ≤20 to 70 years in men respectively to 80 years in women from 10% to 43% respectively 9%–30% which was significantly higher in individuals with a low-compared to a high-performance level. Contrary to these cycle ergometer results the HRPC pattern in the recent study was not different between men and women, and therefore men and women were compiled for overall analyses. Compared to cycle ergometer exercise, the number of non-regular HRPC´s also increased with a linear trend from 3% in 18–26 years old subjects to 15% in the oldest age group between 45–65 years, but values were markedly lower compared to the cycle ergometer study for comparable ages. In line with our results Lucía et al. (2000) showed that only 31% of older master athletes presented a regular downward deflection, with a comparable %VO_2max_ between the HRTP and VT_2._ In this study 69% of subjects presented a linear or even upward deflection which is a greater number compared to our own results for this age group. With respect to the relationship between performance and the HRPC pattern, we also found significant effects and a nearly twice as high percentage of non-regular curves in the low-compared to the high-performance-group. The distribution in age- and performance-groups showed higher relative numbers of non-regular curves in the low-performance groups in every age group (Figure 3), but the difference to the high-performance group was small with only 1.5%–3.9%. This is consistent with the smaller effects of performance compared to age shown in the Chi-squared analysis, which indicated a greater influence of age on the HRPC deflection rather than performance. We may suggest, that HRPC´s in maximal incremental treadmill and cycle ergometer exercise show a similar distribution of the different HRPC patterns, although the number of non-regular curves was clearly lower in the treadmill tests. An S-shaped pattern of the HRPC may be suggested as the regular response to graded exercise independent of the exercise mode.

A reduced β1-receptor sensitivity to the exercise catecholamine response has been suggested as the main reason for the differences in the HRPC pattern (Hofmann et al., 2005). Since the increase in HR with increasing exercise intensity is closely related to the increase in plasma catecholamine concentrations (Chwalbinska-Moneta et al., 1996; White and Raven, 2014), the flattening of HR at maximum intensities is supposed to be caused by a saturation of β1-receptors (Hofmann et al., 2005) although catecholamines continue to increase in this intensity phase (Pokan et al., 1995; Moser et al., 2017) (Figure 1). On the other hand, if β1-receptor sensitivity is somehow reduced, HR increase is blunted in the second phase and increases disproportionally in the third intensity phase of energy metabolism due to an exponential increase of catecholamine levels, exceeding the insensitivity of the receptors (Shah et al., 2019), causing an inverse (upward) deflection of the HRPC (Hofmann et al., 2005). Hofmann et al. (2005) showed this effect in their study blocking β1-receptor sensitivity by a single dose of the β1-selective antagonist bisoprolol in healthy individuals, which reduced the degree of deflection and even changed regular HRPC´s to inverse, revealing a significant association between the response to the antagonist and different HRPC patterns. Women compared to men (Johansson and Hjalmarson, 1988; Turner et al., 1999) and individuals with a high performance level (Lehmann et al., 1984; Hofmann et al., 2005) were shown to have a higher β1-receptor sensitivity. Contrary, aging has been shown to reduce receptor sensitivity (Fleg et al., 1994; Christou and Seals, 2008). These suggested causes for different HRPC patterns were supported by our recent studies in cycling exercise (Birnbaumer et al., 2020). Due to these effects of age and performance on the HRPC, receptor sensitivity can be assumed to be also the main reason for differences in the HRPC deflection in running tests, (Hofmann et al., 2005; Moser et al., 2018; Birnbaumer et al., 2020; Birnbaumer et al., 2021). Several hypotheses have been pursued to explain the differences in the variations of the HRPC patterns (Hofmann and Pokan, 2010), but no model other than the β1-adrenergic receptor hypothesis proved to explain the differences. However, additional running exercise specific factors affecting differences between incremental cycle and treadmill exercise cannot be excluded and need to be explored in further studies. Rosic et al. (2011) for example, addressed the variations in the deflection of the HRPC to the exercise protocol. In their study they investigate the effect of three different protocols on the deflection of the HRPC. However, they did not consider the anticipatory phase at the start of exercise as proposed by Brooke et al. (1968) respectively in one of the protocols the intensity at the beginning of exercise might have been even too high to show such a phase. In our treadmill study with a uniform exercise protocol the HRPC showed three different patterns, in line with results from cycling where also a uniform protocol was applied (Hofmann et al., 1997; Moser et al., 2018; Birnbaumer et al., 2020). At present, the different patterns of the HRPC cannot be directly referred to specific health or risk factors. However, chronic stress independent of its origin downregulates ß1-adrenoceptors, which is suggested to cause non-regular HRPC`s such as shown in patients suffering from type I diabetes (Moser et al., 2018). Any non-regular HRPC or a change in that direction may therefore be an unspecific sign of chronic stress conditions which should be observed.

### 4.2 GXT performance

Our study adds to reference standards for VT_1_ and VT_2_ in men and women across the adult age spectrum (Tables 1, 2). As such standards are lacking for maximum GXT treadmill exercise our data provide valuable information for the practical application as well as about general exercise metrics. As expected, maximal V̇O_2_ and HR were the highest in the youngest age groups in men and women and declined with increasing age. Mean values as well as the age dependent decline were comparable to maximal treadmill reference data from 3,816 healthy men and women published by Loe et al. (2013). Additionally, mean relative values at VT_1_ and VT_2_ for HR and VO_2_ were consistent with the results of other studies (Vucetic et al., 2014; Vainshelboim et al., 2020). Reference values for the first threshold were reported at approximately 63% for %V̇O_2max_ and 75% for %HR_max_ from a large data set of 1,195 (550 women) treadmill tests (Vainshelboim et al., 2020). The second ventilatory threshold was measured at 86% for %V̇O_2max_ and 91% for %HR_max_ in a group of 48 experienced runners (Vucetic et al., 2014).

However, although mean values are very consistent, relative performance values were already shown to have a high variability and therefore function poorly for the prescription of exercise intensity aiming for standardized metabolic stress (Hofmann et al., 2001; Hansen et al., 2019; Iannetta et al., 2019; Meyler et al., 2023). In this regard Iannetta et al. (2019) showed that %HR_max_ at the first and second thresholds from maximal incremental cycle ergometer exercise ranged from 60% to 90% and from 75% to 97%, respectively in a group of 100 healthy adults. %V̇O_2max_ at the thresholds was found between 45% and 74% for the first and between 69% and 96% for the second threshold. Our results show a comparable high variability of 39–86 %V̇O_2max_ and 60–93% HR_max_ at VT_1_ as well as 51%–98% V̇O_2max_ and 70–98% HR_max_ at VT_2_ indicating the need for individual exercise prescription applying thresholds (Meyler et al., 2023).

Variations in %HR_max_ at the second threshold were already shown to be dependent on the HRPC pattern and were found around 83% in non-regular inverse HRPC´s and 90% in regular downward deflecting curves (Hofmann et al., 1997; Hofmann et al., 2001; Hofmann et al., 2005). Comparable to these earlier findings from Hofmann et al. for cycling exercise, mean %HR_max_ at VT_2_ in our treadmill study was significantly lower in participants with a non-regular compared to regular HRPC, but the percentage was markedly higher at 88%. This is since %HR_max_ at VT_2_ ranged between 70% and 95% and a considerable amount of non-regular curves showed a %HR_max_ greater than 90% whereas this range of %HR_max_ was smaller with a higher mean value, namely, between 85% and 98% in the regular HRPC´s. Non-regular curves with an inverse deflection and such a high %HR_max_ presented a steep increase already in the first phase of exercise, rather high HR during the second phase followed by a further increase approaching maximal intensities. This is somehow different to the HRPC´s found in cycle ergometer exercise, were HR was shown to increase steadily but blunted in the second phase in non-regular inverse curves (Hofmann et al., 2005). Poor running economy and technique may be suggested as a reason for such a HR increase disproportionate to the running speed. However, half of the individuals with a non-regular HRPC presented %HR_max_ at VT_2_ between 70% and 89% which increases the likelihood of overestimating exercise intensity in this group prescribing exercise intensity by fixed values of relative intensity according to guidelines (ACSM, 2021) (Hofmann and Tschakert, 2010; Birnbaumer et al., 2020).

### 4.3 Limitations

Compared to our large cycle ergometer study (Birnbaumer et al., 2020) no subjects older than 65 years were included in the treadmill study. As stronger effects were found for the older subjects in the cycle ergometer study, these age groups need to be investigated in an additional study to prove such age effects also for treadmill running exercise. Regarding the conclusion of the cause for different HRPC patterns it is limiting that there are no measurements of the catecholamine levels during the test or long-term stress markers like cortisol. Furthermore, we did not analyze the VO_2_ response regarding the relationship between performance, stroke volume and the HRPC pattern at maximal intensities. However, earlier results showed no differences for maximal power output and oxygen uptake in young healthy trained subjects with regular vs. non-regular HR curves (Hofmann et al., 1994) despite differences in left-ventricular function summarized in Hofmann and Pokan. (2010).

## 5 Conclusion

To our knowledge this is the first study describing the distribution of the HRPC pattern in maximal graded treadmill running exercise. Comparable to incremental cycle ergometer exercise, HRPC´s in treadmill exercise also showed three different patterns, namely, regular downward deflection (91%) and non-regular linear (≈2%) or inverse (≈7%) cases. The number of non-regular curves were shown to be greater in older age or with a lower performance level. The fact that the different HRPC patterns are not limited to cycling exercise gives additional meaning for the assessment of the HRPC´s in incremental exercise. In addition, this study provides absolute and relative reference standards for maximal, VT_1_ and VT_2_ values for treadmill running exercise in men and women across a large age spectrum and as a function of performance.

## Data Availability

The raw data supporting the conclusion of this article will be made available by the authors, without undue reservation.
